# Supplementary material for: Discovery and application of insertion-deletion (INDEL) polymorphisms for QTL mapping of early life-history traits in Atlantic salmon
Source: BMC Genomics. 2010 Mar 8;11:156. doi: 10.1186/1471-2164-11-156 (PMC2838853; doi:10.1186/1471-2164-11-156)
Supplement: Additional file 2 — Information on developed 76 locus single-run INDEL panel in Atlantic salmon. Information on fluorescence labeling, primer concentrations, PCR pooling and links to alignments, INDEL motifs and GENESCAN (Burge and Karlin 1997) predictions of genes/exons are available in html format. [file 1471-2164-11-156-S2.ZIP › Additionalfile2/table21188.html]

Contig Alignment

```
Base	A B C D   
1	. C . .    
2	. C . .    
3	. A . .    
4	. G . .    
5	. C . .    
6	. T . .    
7	. A . .    
8	. T . .    
9	. C . .    
10	. A . .    
11	. A . .    
12	. A . .    
13	. T . .    
14	. C . .    
15	. C . .    
16	. T . .    
17	. T . .    
18	. G . .    
19	. C . .    
20	. C . .    
21	. C . .    
22	. C . .    
23	. A . .    
24	. A . .    
25	T T T .    
26	C C C .    
27	G G G .    
28	G G G .    
29	T G T .  1	 transversion  
30	G G G .    
31	G G G .    
32	A A A .    
33	C C C .    
34	A A A .    
35	T T T .    
36	T T T .    
37	A A A .    
38	C C C .    
39	A A A .    
40	C C C .    
41	A A A .    
42	A A A .    
43	A A A .    
44	A A A .    
45	T T T .    
46	G G G .    
47	T T T .    
48	G G G .    
49	T T T .    
50	T T T .    
51	A A A .    
52	G G G .    
53	C C C .    
54	T T T .    
55	A A A .    
56	C C C .    
57	T T T .    
58	T T T .    
59	A A A .    
60	G G G .    
61	C C C .    
62	T T T .    
63	T T T .    
64	T T T .    
65	C C C .    
66	A A A .    
67	C C C .    
68	T T T .    
69	G G G .    
70	A A A .    
71	C C C .    
72	T T T .    
73	T T T .    
74	T T T .    
75	T T T .    
76	C C C .    
77	T T T .    
78	C C C .    
79	T T T .    
80	T T T .    
81	A A A .    
82	T T T .    
83	G G G .    
84	G G G .    
85	C C C .    
86	A A A .    
87	C C C .    
88	A A A .    
89	C C C .    
90	C C C .    
91	C C C .    
92	G G G .    
93	A A A .    
94	T T T .    
95	G G G .    
96	T T T .    
97	G G G .    
98	G G G .    
99	T T T .    
100	A A A .    
101	A A A .    
102	A A A .    
103	C C C .    
104	A A A .    
105	A A A .    
106	A A A .    
107	G G G .    
108	C C C .    
109	C C C .    
110	T T T .    
111	T T T .    
112	T T T .    
113	A A A .    
114	T T T .    
115	T T T .    
116	A A A .    
117	A A A .    
118	C C C .    
119	C C C .    
120	A A A .    
121	G G G .    
122	T T T .    
123	C C C .    
124	G G G .    
125	C C C .    
126	C C C .    
127	A A A .    
128	T T T .    
129	C C C .    
130	A A A .    
131	C C C .    
132	A A A .    
133	T T T .    
134	C C C .    
135	C C C .    
136	A A A .    
137	C C C .    
138	C C C .    
139	T T T .    
140	A A A .    
141	G G G .    
142	C C C .    
143	A A A .    
144	C C C .    
145	T T T .    
146	G G G .    
147	A A A .    
148	G G G .    
149	T T T .    
150	C C C .    
151	G G G .    
152	A A A .    
153	C C C .    
154	C C C .    
155	C C C .    
156	C C C .    
157	T T T .    
158	C C C .    
159	T - T .  1	 indel  
160	G - G .  1	 indel  
161	A A A .    
162	C C C .    
163	T T T .    
164	C C C .    
165	T T T .    
166	G G G .    
167	T T T .    
168	A A A .    
169	A A A .    
170	C C C .    
171	T T T .    
172	A A A .    
173	C C C .    
174	T T T .    
175	C C C .    
176	A A A .    
177	G G G .    
178	T T T .    
179	C C C .    
180	A A A .    
181	T T T .    
182	G G G .    
183	G G G .    
184	A A A .    
185	C C C .    
186	A A A .    
187	A A A .    
188	A A A .    
189	G G G .    
190	G G G .    
191	T T T .    
192	T T T .    
193	T T T .    
194	G G G .    
195	G G G .    
196	T T T .    
197	A A A .    
198	C C C .    
199	C C C .    
200	A A A .    
201	G G G .    
202	G G G .    
203	T T T .    
204	A A A .    
205	G G G .    
206	G G G .    
207	A A A .    
208	C C C .    
209	C C C .    
210	A A A .    
211	G G G .    
212	T T T .    
213	T T T .    
214	C C C .    
215	G G G .    
216	G G G .    
217	T T T .    
218	C C C .    
219	T T T .    
220	C C C .    
221	A A A .    
222	G G G .    
223	A A A .    
224	G G G .    
225	A A A .    
226	T T T .    
227	G G G .    
228	G G G .    
229	A A A .    
230	G G G .    
231	G G G .    
232	G G G .    
233	G G G .    
234	A A A .    
235	T T T .    
236	A A A .    
237	T T T .    
238	G G G .    
239	T T T .    
240	G G G .    
241	T T T .    
242	G G G .    
243	A A A .    
244	T T T .    
245	A A A .    
246	C C C .    
247	C C C .    
248	A A A .    
249	G G G .    
250	A A A .    
251	T T T .    
252	A A A .    
253	T T T .    
254	G G G .    
255	A A A .    
256	G G G .    
257	T T T .    
258	G G G .    
259	G G G .    
260	C C C .    
261	A A A .    
262	T T T .    
263	C C C .    
264	A A A .    
265	A A A .    
266	A A A .    
267	T T T .    
268	C C C .    
269	G G G .    
270	G G G .    
271	A A A .    
272	A A A .    
273	C C C .    
274	A A A .    
275	C C C .    
276	C C C .    
277	C C C .    
278	G G G .    
279	G G G .    
280	T T T .    
281	T T T .    
282	G G G .    
283	G G G .    
284	G G G .    
285	A A A .    
286	G G G .    
287	T T T .    
288	G G G .    
289	C C C .    
290	G G G .    
291	G G G .    
292	A A A .    
293	C C C .    
294	A A A .    
295	A A A .    
296	C C C .    
297	A A A .    
298	C C C .    
299	A A A .    
300	G G G .    
301	G G G .    
302	T T T .    
303	G G G .    
304	C C C .    
305	A A A .    
306	C C C .    
307	A A A .    
308	G G G .    
309	A A A .    
310	A A A .    
311	C C C .    
312	G G G .    
313	T T T .    
314	G G G .    
315	G G G .    
316	C C C .    
317	C C C .    
318	A A A .    
319	T T T .    
320	G G G .    
321	G G G .    
322	G G G .    
323	G G T .  1	 transversion  
324	A A A .    
325	T T T .    
326	C C C .    
327	A A A .    
328	A A A .    
329	G G G .    
330	T T T .    
331	T T T .    
332	C C C .    
333	A A A .    
334	T T T .    
335	T T T .    
336	C C C .    
337	T T T .    
338	G G G .    
339	C C C .    
340	C C C .    
341	C C C .    
342	A A A .    
343	A A A .    
344	C C C .    
345	C C C .    
346	G G G .    
347	C C C .    
348	T T T .    
349	T T T .    
350	C C C .    
351	G G G .    
352	A A A .    
353	T T T .    
354	A A A .    
355	T T T .    
356	G G G .    
357	A A A .    
358	A A A .    
359	C C C .    
360	G G G .    
361	T T T .    
362	G G G .    
363	T T T .    
364	G G G .    
365	C C C C    
366	T T T T    
367	C C C C    
368	A A A A    
369	A A A A    
370	G G G G    
371	A A A A    
372	T T T T    
373	T T T T    
374	C C C C    
375	G G G G    
376	T T T T    
377	C C C C    
378	A A A A    
379	A A A A    
380	G G G G    
381	T T T T    
382	C C C C    
383	A A A A    
384	C C C C    
385	T T T T    
386	C C C C    
387	A A A A    
388	A A A A    
389	C C C C    
390	. G G G    
391	. A A A    
392	. G G G    
393	. G G G    
394	. A A A    
395	. G G G    
396	. G G G    
397	. A A A    
398	. C C C    
399	. A A A    
400	. G G G    
401	. C C C    
402	. A A A    
403	. A A A    
404	. A A A    
405	. A A A    
406	. A A A    
407	. C C C    
408	. A A A    
409	. T T T    
410	. C C C    
411	. C C C    
412	. A A A    
413	. G G G    
414	. G G G    
415	. A A A    
416	. C C C    
417	. C C C    
418	. A A A    
419	. G G G    
420	. G G G    
421	. T T T    
422	. C C C    
423	. A A A    
424	. A A A    
425	. C C C    
426	. T T T    
427	. C C C    
428	. C C C    
429	. G G G    
430	. A A A    
431	. T T T    
432	. T T T    
433	. T T T    
434	. G G G    
435	. G G G    
436	. A A A    
437	. G G G    
438	. G G G    
439	. T T T    
440	. G G G    
441	. G G G    
442	. C C C    
443	. T T T    
444	. T T T    
445	. C C C    
446	. T T T    
447	. G G G    
448	. T T T    
449	. T T T    
450	. T T T    
451	. T T T    
452	. A A A    
453	. T T T    
454	. T T T    
455	. T T T    
456	. A A A    
457	. A A A    
458	. A A A    
459	. G G G    
460	. C C C    
461	. C C T  1	 transition  
462	. G G G    
463	. A A A    
464	. G G G    
465	. T T T    
466	. G G G    
467	. T T T    
468	. A A A    
469	. A A A    
470	. T T T    
471	. A A A    
472	. T T T    
473	. T T T    
474	. C C C    
475	. A A A    
476	. G G G    
477	. A A A    
478	. C C C    
479	. G G G    
480	. T T T    
481	. C C C    
482	. A A A    
483	. C C C    
484	. C C C    
485	. C C C    
486	. T T T    
487	. C C C    
488	. A A A    
489	. C C C    
490	. C C C    
491	. T T T    
492	. G G G    
493	. G G G    
494	. G G G    
495	. A A A    
496	. T T T    
497	. A A A    
498	. C C C    
499	. A A A    
500	. A A A    
501	. G G G    
502	. T T T    
503	. G G G    
504	. C C C    
505	. G G G    
506	. T T T    
507	. C C C    
508	. A A A    
509	. T T T    
510	. G G G    
511	. T T T    
512	. T T T    
513	. T T T    
514	. A A A    
515	. T T T    
516	. A A A    
517	. C C C    
518	. C C C    
519	. C C C    
520	. C C C    
521	. G G G    
522	. T T T    
523	. C C C    
524	. C C C    
525	. A A A    
526	. C C C    
527	. C C C    
528	. A A A    
529	. C C C    
530	. C C C    
531	. A A A    
532	. A A A    
533	. A A A    
534	. C C C    
535	. A A A    
536	. C C C    
537	. T T T    
538	. T T T    
539	. C C C    
540	. T T T    
541	. C C C    
542	. T T T    
543	. C C C    
544	. C C C    
545	. C C C    
546	. A A A    
547	. T T T    
548	. C C C    
549	. A A A    
550	. A A A    
551	. . A A    
552	. . C C    
553	. . A A    
554	. . G G    
555	. . T T    
556	. . C C    
557	. . G G    
558	. . A A    
559	. . C C    
560	. . A A    
561	. . A A    
562	. . C C    
563	. . A A    
564	. . C C    
565	. . T T    
566	. . C C    
567	. . A A    
568	. . . C    
569	. . . C    
570	. . . A    
571	. . . A    
572	. . . C    
573	. . . A    
574	. . . A    
575	. . . G    
576	. . . C    
577	. . . A    
578	. . . C    
579	. . . C    
580	. . . G    
581	. . . T    
582	. . . G    
583	. . . G    
584	. . . T    
585	. . . A    
586	. . . A    
587	. . . C    
588	. . . G    
589	. . . A    
590	. . . G    
591	. . . G    
592	. . . T    
593	. . . G    
594	. . . T    
595	. . . C    
596	. . . C    
597	. . . T    
598	. . . C    
599	. . . C    
600	. . . A    
601	. . . C    
602	. . . T    
603	. . . C    
604	. . . C    
605	. . . T    
606	. . . C    
607	. . . T    
608	. . . T    
609	. . . C    
610	. . . T    
611	. . . G    
612	. . . G    
613	. . . T    
614	. . . C    
615	. . . C    
616	. . . A    
617	. . . C    
618	. . . T    
619	. . . G    
620	. . . T    
621	. . . A    
622	. . . A    
623	. . . G    
624	. . . T    
625	. . . A    
626	. . . C    
627	. . . C    
628	. . . T    
629	. . . G    
630	. . . A    
631	. . . G    
632	. . . C    
633	. . . T    
634	. . . C    
635	. . . A    
636	. . . T    
637	. . . C    
638	. . . T    
639	. . . T    
640	. . . A    
641	. . . A    
642	. . . A    
643	. . . C    
644	. . . T    
645	. . . A    
646	. . . A    
647	. . . A    
648	. . . A    
649	. . . G    
650	. . . A    
651	. . . T    
652	. . . G    
653	. . . G    
654	. . . T    
655	. . . T    
656	. . . T    
657	. . . C    
658	. . . T    
659	. . . T    
660	. . . T    
661	. . . G    
662	. . . T    
663	. . . C    
664	. . . C    
665	. . . A    
666	. . . A    
667	. . . T    
668	. . . G    
669	. . . T    
670	. . . A    
671	. . . C    
672	. . . A    
673	. . . C    
674	. . . A    
675	. . . G    
676	. . . A    
677	. . . C    
678	. . . T    
679	. . . T    
680	. . . A    
681	. . . C    
682	. . . A    
683	. . . A    
684	. . . C    
685	. . . T    
686	. . . T    
687	. . . G    
688	. . . T    
689	. . . C    
690	. . . T    
691	. . . T    
692	. . . C    
693	. . . T    
694	. . . G    
695	. . . C    
696	. . . T    
697	. . . T    
698	. . . T    
699	. . . A    
700	. . . T    
701	. . . T    
702	. . . C    
703	. . . C    
704	. . . A    
705	. . . A    
706	. . . C    
707	. . . C    
708	. . . C    
709	. . . C    
710	. . . T    
711	. . . C    
712	. . . T    
713	. . . G    
714	. . . A    
715	. . . A    
716	. . . A    
717	. . . G    
718	. . . A    
719	. . . T    
720	. . . A    
721	. . . A    
722	. . . G    
723	. . . A    
724	. . . G    
725	. . . G    
726	. . . T    
727	. . . C    
728	. . . G    
729	. . . G    
730	. . . A    
731	. . . A    
732	. . . C    
733	. . . A    
734	. . . G    
735	. . . T    
736	. . . G    
737	. . . G    
738	. . . G    
739	. . . C    
740	. . . A    
741	. . . G    
742	. . . C    
743	. . . T    
744	. . . A    
745	. . . T    
746	. . . A    
747	. . . T    
748	. . . G    
749	. . . A    
750	. . . C    
751	. . . G    
752	. . . G    
753	. . . C    
754	. . . T    
755	. . . T    
756	. . . T    
757	. . . C    
758	. . . G    
759	. . . T    
760	. . . C    
761	. . . C    
762	. . . T    
763	. . . G    
764	. . . G    
765	. . . A    
766	. . . T    
767	. . . T    
768	. . . T    
769	. . . G    
770	. . . T    
771	. . . C    
772	. . . A    
773	. . . C    
774	. . . A    
775	. . . T    
776	. . . C    
777	. . . A    
778	. . . C    
779	. . . T    
780	. . . T    
781	. . . A    
782	. . . G    
783	. . . C    
784	. . . C    
785	. . . T    
786	. . . C    
787	. . . A    
788	. . . A    
789	. . . A    
790	. . . C    
791	. . . A    
792	. . . T    
793	. . . G    
794	. . . A    
795	. . . C    
796	. . . T    
797	. . . A    
798	. . . C    
799	. . . A    
800	. . . A    
801	. . . C    
802	. . . A    
803	. . . C    
804	. . . A    
805	. . . A    
806	. . . A    
807	. . . A    
808	. . . C    
809	. . . A    
810	. . . T    
811	. . . A    
812	. . . C    
813	. . . C    
814	. . . T    
815	. . . G    
816	. . . A    
817	. . . A    
818	. . . T    
819	. . . C    
820	. . . G    
821	. . . A    
822	. . . A    
823	. . . C    
824	. . . A    
825	. . . T    
826	. . . T    
827	. . . G    
828	. . . A    
829	. . . A    
830	. . . T    
831	. . . G    
832	. . . A    
833	. . . T    
834	. . . G    
835	. . . G    
836	. . . C    
837	. . . T    
838	. . . G    
839	. . . A    
840	. . . T    
841	. . . G    
842	. . . A    
843	. . . T    
844	. . . T    
845	. . . G    
846	. . . A    
847	. . . C    
848	. . . C    
849	. . . T    
850	. . . C    
851	. . . T    
852	. . . G    
853	. . . A    
854	. . . C    
855	. . . G    
856	. . . T    
857	. . . C    
858	. . . T    
859	. . . T    
860	. . . T    
861	. . . C    
862	. . . C    
863	. . . A    
864	. . . G    
865	. . . C    
866	. . . T    
867	. . . C    
868	. . . T    
869	. . . G    
870	. . . T    
871	. . . C    
872	. . . C    
873	. . . A    
874	. . . T    
875	. . . T    
876	. . . C    
877	. . . A    
878	. . . C    
879	. . . C    
880	. . . A    
881	. . . G    
882	. . . C    
883	. . . T    
884	. . . A    
885	. . . G    
886	. . . C    
887	. . . A    
888	. . . G    
889	. . . C    
890	. . . A    
891	. . . C    
892	. . . A    
893	. . . G    
894	. . . G    
895	. . . G    
896	. . . T    
897	. . . G    
898	. . . A    
899	. . . A    
900	. . . A    
901	. . . T    
902	. . . G    
903	. . . G    
904	. . . T    
905	. . . T    
906	. . . T    
907	. . . T    
908	. . . C    
909	. . . C    
910	. . . T    
911	. . . G    
912	. . . G    
913	. . . C    
914	. . . C    
915	. . . A    
916	. . . G    
917	. . . C    
918	. . . A    
919	. . . G    
920	. . . G    
921	. . . A    
922	. . . T    
923	. . . T    
924	. . . G    
925	. . . A    
926	. . . A    
927	. . . C    
928	. . . A
```
